# CLEC9A modulates macrophage-mediated neutrophil recruitment in response to heat-killed *Mycobacterium tuberculosis* H37Ra

**DOI:** 10.1371/journal.pone.0186780

**Published:** 2017-10-24

**Authors:** An-Chieh Cheng, Kuang-Yao Yang, Nien-Jung Chen, Tsui-Ling Hsu, Ruwen Jou, Shie-Liang Hsieh, Ping-Hui Tseng

**Affiliations:** 1 Institute of Biochemistry and Molecular Biology, School of Life Science, National Yang-Ming University, Taipei, Taiwan; 2 Institute of Emergency and Critical Care Medicine, School of Medicine, National Yang-Ming University, Taipei, Taiwan; 3 Department of Chest Medicine, Taipei Veterans General Hospital, Taipei, Taiwan; 4 Infection and Immunity Research Center, National Yang-Ming University, Taipei, Taiwan; 5 Institute of Microbiology and Immunology, School of Life Science, National Yang-Ming University, Taipei, Taiwan; 6 Genomics Research Center, Academia Sinica, Taipei, Taiwan; 7 Taiwan Centers for Disease Control, Taipei, Taiwan; Fundació Institut d’Investigació en Ciències de la Salut Germans Trias i Pujol, Universitat Autònoma de Barcelona, SPAIN

## Abstract

Tuberculosis is a fatal human infectious disease caused by *Mycobacterium tuberculosis* (*M*. *tuberculosis*) that is prevalent worldwide. Mycobacteria differ from other bacteria in that they have a cell wall composed of specific surface glycans that are the major determinant of these organisms' pathogenicity. The interaction of *M*. *tuberculosis* with pattern recognition receptors (PRRs), in particular C-type lectin receptors (CLRs), on the surface of macrophages plays a central role in initiating innate and adaptive immunity, but the picture as a whole remains a puzzle. Defining novel mechanisms by which host receptors interact with pathogens in order to modulate a specific immune response is an area of intense research. In this study, based on an *in vitro* lectin binding assay, CLEC9A (DNGR-1) is identified as a novel CLR that binds with mycobacteria. Our results with CLEC9A-knocked down cells and a CLEC9A-Fc fusion protein as blocking agents show that CLEC9A is involved in the activation of SYK and MAPK signaling in response to heat-killed *M*. *tuberculosis* H37Ra treatment, and it then promotes the production of CXCL8 and IL-1β in macrophages. The CXCL8 and IL-1β secreted by the activated macrophages are critical to neutrophil recruitment and activation. In a *in vivo* mouse model, when the interaction between CLEC9A and H37Ra is interfered with by treatment with CLEC9A-Fc fusion protein, this reduces lung inflammation and cell infiltration. These findings demonstrate that CLEC9A is a specialized receptor that modulates the innate immune response when there is a mycobacterial infection.

## Introduction

Tuberculosis (TB) is a fatal human infectious disease that occurs worldwide and is caused by a bacterium called *Mycobacterium tuberculosis* (*M*. *tuberculosis*) [1]. *M*. *tuberculosis* is an aerobic, and slow growing mycobacterium that divides every 15 to 20 hours and is a TB pathogen. The outcome of *M*. *tuberculosis* infection can range from early asymptomatic clearance through to latent infection and thence to the clinical disease [2]. The mechanism for asymptomatic clearance is still unknown. On the other hand, it is known that an immune response is triggered against *M*. *tuberculosis* infection. After inhalation, *M*. *tuberculosis* is engulfed by alveolar macrophages and dendrite cells (DCs), which initiates the innate response, and present antigens for T cell differentiation [3]. Antigen-specific T cells secrete IFNγ and TNF to activate macrophages and induce granuloma formation around the infected macrophages. Neutrophils accumulate during mycobacterial infection, but the role of neutrophils in the pathogenesis of *M*. *tuberculosis* infection in humans is not completely understood [4]. Although understanding the immunological mechanisms leading to the various outcomes of *M*. *tuberculosis* infection remains problematic, there is no doubt that the interaction of mycobacteria with the innate immune system plays a central role in the events of pathogenesis.

The mycobacterial cell wall is thicker than that of other bacteria and contains peptidoglycan, polysaccharides (arabinogalactan) and mycolic acids from inside to outside, as well as inserts of lipoarabinomannan (LAM), and phosphatidylinositol mannoside (PIM) [5]. It has been demonstrated that the surface glycans are the major determinant of *M*. *tuberculosis* pathogenicity [6]. Innate immunity is able to recognize mycobacteria via various pattern recognition receptors (PRRs) that trigger downstream signal activation and induces the production of cytokines and chemokines, which then regulate the infection [7]. Currently, four different classes of PRR families have been identified, namely the toll-like receptors (TLRs), the nod-like receptors (NLRs), the c-type lectin receptors (CLRs) and the RIG-I-like receptors (RLRs) [8]. TLRs and NLRs have been shown to be involved in mycobacterial infection and recognition [9]. TLR2 and TLR4 are able to recognize LAM, lipomannan (LM) and PIM on the cell wall of mycobacteria [10]. The 19-KDa lipoprotein of *M*. *tuberculosis* is able to activate macrophages via TLR2 [11]. Moreover, the DNA of mycobacteria includes CpG motifs and these are able to activate TLR9 [12]. Mice that lack MyD88, a critical adaptor in TLR signaling, are highly susceptible to *M*. *tuberculosis* infection [13]. NOD2-deficient mice have a defect in cytokine production when there is a mycobacteria infection [14]. An earlier report has also shown that mycobacteria prevent the activation of the inflammasome and inhibit IL-1β processing [15].

As the major PRRs involved in carbohydrate recognition, CLRs should be the key to interactions with mycobacteria *in vivo* [16]. It is known that the mannose receptor (MRC1 or MRC2) recognizes LAM and mediates the phagocytosis of mycobacteria [17]. In addition, CLEC4L (DC-SIGN) recognizes LAM of mycobacteria and modulates the functioning of DCs [18, 19], while CLEC7A (Dectin-1) is able to induce TNF, IL-6 and IL-12 production in the presence of mycobacteria [20], and CLEC6A (Dectin-2) recognizes LAM to induce nitric oxide (NO) production [21]. Finally, trehalose-6,6’-dimycolate (TDM), a glycolipid on the mycobacterial cell wall, is able to bind with CLEC4E (Mincle) or with CLEC4D (MCL), which then leads to modulation of macrophage activation [22, 23].

Based on an *in vitro* lectin binding assay [24], CLEC9A (DNGR-1) has been identified as a novel candidate protein that seems to be able to bind with heat-killed or viable mycobacteria. It has been suggested that CLEC9A is highly expressed in DCs, but it is also known to be expressed in myeloid-lineage cells, where it functions as an endocytotic receptor [25–28]. However, little is known about the involvement of CLEC9A in response to *M*. *tuberculosis*. In the above context, it should be noted that defining the mechanisms by which mycobacterial components and their distinct receptors regulate the immune response in humans and animals is an area of intense research.

Here, we have focused on investigating the role of CLEC9A in macrophages using heat-killed mycobacteria as the “infective' agent and have demonstrated that CLEC9A is involved in SYK and MAPK activation and is able to selectively promote downstream CXCL8 and IL-1β production in response to *M*. *tuberculosis* H37Ra. Furthermore, the recruitment of neutrophils *in vitro* and *in vivo* seems to be modulated by CLEC9A. Our results suggest that CLEC9A, a novel CLR that can interact with mycobacteria, is able to selectively regulate signal activation and cytokine production and this, in turn, allows modulation of the innate immune response during *M*. *tuberculosis* infection.

## Materials and methods

### Ethics statement

The use of human samples and animals was in accordance with national approved law (Human Subjects Research Act and Animal Protection Act) and institutional approved guidelines. Human monocytes and neutrophils were obtained from healthy donors approved by the Institutional Review Board of National Yang-Ming University (IRB #YM102026E). Written informed consent was obtained from donors before they entered the study protocol. All animal studies were performed according to the animal study protocol approved by the Institutional Animal Care and Use Committee (IACUC) of National Yang-Ming University (IACUC #1031203) and the recommendation of “A Guidebook for Care and Use of Laboratory Animals” (Third Edition). All efforts were made to minimize animal discomfort and suffering.

### Reagents and antibodies

SYK inhibitor, BAY 61–3606, Phorbol 12-myristate 13-acetate (PMA), Fluorescein isothiocyanate isomer 1 and LPS were obtained from Sigma. The following commercial antibodies were used. Anti-Syk (sc-1240), anti-IKKα/β (sc-7607), anti-JNK (sc-7345), anti-p38 (sc-728), and anti-β-actin (sc-69879) were purchased from Santa Cruz. Anti-p-Syk (2711), anti-p-IKKα/β (9246), anti-p-JNK (9251), anti-p-p38 (9211) were purchased from Cell Signaling. Anti-CLEC9A (ab79661) were purchased from Abcam. Goat F(ab')_2_ Anti-Human IgG-PE (2042–09) was obtained from Southern Biotech. Goat F(ab')_2_ Anti-Rabbit IgG-Alexa Fluor 594 (A11072) was obtained from Invitrogen.

### Bacteria

Heat-killed *Mycobacterium tuberculosis* Beijing modern sublineage strain and *Mycobacterium bovis* BCG (Pasteur) were provided by the Centers for Disease Control, Taiwan. Heat-inactivated *Mycobacterium tuberculosis* H37Ra (Cat#231141) was purchased from BD Biosciences. *Mycobacterium kansasii* was obtained from Dr. Hsin-Chih Lai (Chang-Kung University, Taiwan), and grown at 35°C on Middlebrook 7H11 agar medium (Difco Laboratories) supplemented with 10% OADC (oleic acid, albumin, dextrose, catalase; Becton Dickinson). Heat-killed bacteria were prepared by incubation for 30 min at 80°C, and loss of viability was confirmed by plating on 7H11 plates [29]. FITC-labeled mycobacteria were obtained by incubation of H37Ra with 0.5 mg/ml Fluorescein isothiocyanate isomer 1 in PBS, pH 7.4 at room temperature for 1 hour. The FITC-labeled H37Ra were washed three times to remove any unbound FITC. Sufficient labeling of the cells were then checked by fluorescence microscopy.

### Cell culture and treatment

HEK293T, THP-1 and HL-60 cells were obtained from American Type Culture Collection (ATCC). HEK293T cells were grown in Dulbecco’s modified Eagle’s medium (DMEM) containing 10% fetal bovine serum (FBS) and 1% (v/v) penicillin-streptomycin solution. THP-1 cells were cultured in RPMI1640 containing 10% FBS, 1 mM sodium pyruvate, 0.05 mM 2-mercaptoethanol and 1% (v/v) penicillin-streptomycin solution. HL-60 cells were cultured in RPMI1640 containing 20% FBS and 1% (v/v) penicillin-streptomycin solution. HL-60 were differentiated into neutrophil-like cells by treating the cells with 1.25% DMSO for 7 days [30]. RPMI1640, DMEM, sodium pyruvate and penicillin-streptomycin solution were from obtained Biological Industries. FBS was purchased from Invitrogen. The FreeStyleTM 293-F cells (Invitrogen) were cultured by following the instruction provided by the manufacturer. Briefly, the cells were cultured in FreeStyle 293 Expression Medium (Invitrogen) using Erlenmeyer flasks. They were incubated at 37°C in an incubator containing 8% CO_2_ on an orbital shaker rotating at 125 rpm. For H37Ra stimulation, 1×10^5^ THP-1 cells were washed once with PBS, and then incubated with 50 μg (about 5×10^5^) H37Ra in suspension for the indicated times. For receptor-Fc fusion protein blocking, 5×10^5^ H37Ra were pre-incubated with hIgG1-Fc or with CLEC9A-Fc fusion protein (1 or 5 μg) using binding buffer containing 1% BSA, 2 mM Ca^2+^, and 2 mM Mg^2+^ in TBS, pH 7.4, for 1.5 hours. The cells were then washed two times with PBS before treatment.

### Plasmids

pcDNA3.1 CLEC9A.Fc was provided by Dr. Shie-Liang Hsieh [24]. The lentivirus-based specific *CLEC9A* gene knockdown constructs in the pLKO_TRC1 or pLKO_TRC2 vector, and the packaging plasmids, pCMVΔ 8.91 and pMD.G were from the National RNAi Core Facility (Institute of Molecular Biology/Genomics Research Center, Academia Sinica, Taiwan). The target sequences for 3′ UTR region of human CLEC9A were as follow: 5’-CTGGGACTCAATAATACACTT-3’ and 5’-CACACCGTCCAGATTTCATTT-3’.

### *In vitro* lectin binding assay

The *in vitro* lectin binding assay, which used flow cytometry, was modified from that described in a previous report [31]. Briefly, the lectin receptor-Fc fusion proteins (1 μg/ml) were incubated with 5×10^5^ *M*. *tuberculosis* Beijing strain bacteria, *5*×*10*^*5*^ *M*. *bovis* BCG bacteria or 5×10^5^ *M*. *tuberculosis* H37Ra bacteria using binding buffer containing 1% BSA, 2 mM Ca^2+^, and 2 mM Mg^2+^ in TBS, pH 7.4 for 30 min at room temperature. After removing the supernatant by centrifugation and washing the pellet with TBS for three times, the samples were incubated with Goat F(ab')2 Anti-Human IgG-PE, washed and analyzed by flow cytometry.

### Confocal microscopy

THP-1 cells (1×10^4^) were seeded onto coverslips and cultured in the presence 100 ng/ml PMA for 24 hours to induce differentiation of macrophage-like cells [32]. The cells were then incubated with 5×10^4^ FITC-H37Ra in binding buffer for 1 hour at 25°C, washed three times with PBS, fixed with methanol for 30 min at 4°C and blocked with 5% BSA for 1 hour at 25°C. For immunostaining, the cells were incubated with CLEC9A antibody for 24 hours at 4°C, washed, and incubated with Goat Anti-Rabbit Alexa Fluor 594 for 1 hour at 25°C. For nuclei staining, the cells were stained with DAPI (4′,6-diamidino-2-phenylindole; Sigma-Aldrich). After mounting, images were obtained using a laser scanning confocal microscope (LSM 700; Zeiss).

### Lentivirus production and infection

The HEK293T cells were transfected with the lentivirus-based constructs along with the packaging plasmids, pCMVΔ 8.91 and pMD.G using T-pro NTRII (T-Pro Biotechnology, Taiwan). Virus-containing medium was collected at 48 and 72 hours post-transfection. THP-1 cells were infected with lentivirus-containing medium at a multiplicity of infection (MOI) of 10 to 25 in the presence of 5 mg/ml polybrene (Sigma). After 24 hours, the virus-containing medium was replaced with selection medium containing 5 mg/ml puromycin (EMD Millipore). After cell growth was stable, cells were used in subsequent experiments.

### Immunoblotting

Cells were lysed in ice-cold RIPA lysis buffer containing 50 mM Tris-HCl, pH 8.0, 150 mM NaCl, 1% NP40, 0.5% deoxycholate, 0.1% SDS, 1 mM PMSF, 1 mM Na_3_VO_4_ and 1 mM NaF. After centrifugation, the proteins in each cell extract were separated by SDS-PAGE and analyzed by immunoblotting. The membranes were then probed with the indicated antibodies. The blots were visualized using a chemiluminescence reagent (Thermo, Rockford, IL) and exposure of a X-ray film (Fujifilm).

### Quantitative real-time PCR analysis

Total RNA was extracted from cells using TRIzol (Invitrogen), and cDNA complementary DNA (cDNA) was synthesized using a iScript cDNA synthesis kit (Bio-Rad). The amount of mRNA present in each sample were quantified by real-time quantitative polymerase chain reaction (RT-qPCR) using the StepOnePlus system (Applied Biosystems). The mRNA expression level of each target gene was normalized against GAPDH expression in the same cells. The primers for Q-PCR are shown as below: human TNF: 5’-CGAGTGACAAGCCTGTAGC for forward sequence, 5’-GGTGTGGGTGAGGAGCACAT-3’ for reverse sequence; human IL-6: 5’-AAATGCCAGCCTGCTGACGAAG-3’ for forward sequence, and 5’ AACAACAATCTGAGGTGCCCATGCTAC-3’ for reverse sequence; IL-10: 5’-GATGCCTTCAGCAGAGTGAA-3’ for forward sequence, and 5’-GCAACCCAGGTAACCCTTAAA-3’ for reverse sequence; human IL-12 p40: 5’-GCTGGGAGTACCCTGACAC-3’ for forward sequence, and 5’-TTGGGTCTATTCCGTTGTGT-3’ for reverse sequence; human IL-1β: 5’-ACGAATCTCCGACCACCACT-3’ for forward sequence, and 5’-CCATGGCCACAACAACTGAC-3’ for reverse sequence; human CXCL8: 5’-CTGGCCGTGGCTCTCTTG-3’ for forward sequence, and 5’-CCTTGGCAAAACTGCACCTT-3’ for reverse sequence; human CLEC9A: 5’-CCAAGTCTGTGGATACGTGAAA-3’ for forward sequence, and 5’-GAGGATCTCAACGCATACTTCTC-3’ for reverse sequence; human MMP9: 5’-CAACATCACCTATTGGATCC-3’ for forward sequence, and 5’-CGGGTGTAGAGTCTCTCGCT-3’ for reverse sequence; human GAPDH: 5’-GTCATCATATTTGGCAGGTT-3’ for forward sequence, and 5’-GAAGGACTCATGACCACAGT-3’ for reverse sequence.

### Enzyme-linked immunosorbent assay

THP-1 cells were incubated with mycobacteria H37Ra for 8 hours, next the cytokine-containing medium was collected and the amount of various proteins present was then measured using a human TNF and CXCL8 DuoSet ELISA Development kit (R&D, Minneapolis, MN). In the animal study, bronchoalveolar lavage (BAL) fluid was collected from the mice and the amount of protein present was also measured using a mouse TNF and KC DuoSet ELISA Development kit.

### Expression and purification of Fc-tagged recombinant fusion proteins

CLEC9A-Fc fusion protein was produced using the FreeStyle 293 Expression System (Invitrogen) by following the manufacture’s instruction. The medium containing CLEC9A-Fc protein was purified by passing it through a 50KD size exclusion column (Millipore) and the supernatant containing proteins with a molecular weight over 50KD, which included the CLEC9A-Fc protein, was collected. The presence of the purified CLEC9A-Fc protein was confirmed by immunoblotting, and quantified by protein assay dye (Bio-red).

### Isolation of human neutrophil and peripheral blood mononuclear cell (PBMC)

Human neutrophils were isolated from the whole blood of healthy human donors using density gradient centrifugation and hypotonic lysis of any red blood cells that remained [33]. The peripheral blood mononuclear cells (PBMCs) were isolated from the whole blood of healthy human donors by standard density-gradient centrifugation using Ficoll-Paque [34].

### Neutrophil migration assay

The migration assays were conducted in a modified 24-well (3.0 mm) Transwell system (Corning Costar #3415) [35]. HL-60D cells or human neutrophils (2×10^5^ cells) were added to the upper well and conditioned medium from THP-1 cells with or without mycobacteria H37Ra treatment was placed in the lower well. For the positive control, 1 ng/ml MIP-2 (R&D Systems) was placed in the lower well to act as a chemotactic stimulus. After 2 hours incubation, the migrated cells present in the lower well were collected and transferred to microscope slides by Cytospin. The cells in microscope slides were fixed with 10% formalin, stained with DAPI, and counted using five random microscopic fields.

### Animal model—Intratracheal injection

C57BL/6 mice, male, aged 6–7 weeks, were obtained from the National Laboratory Animal Breeding and Research Center (Taipei, Taiwan). All mice were maintained in the specific pathogen-free facility of the Laboratory Animal Research Center at Yang-Ming University under a 12 hour light/dark cycle (light from 7 am to 7 pm) at 22±1°C, with unrestricted access to sterilized food and water. Veterinary care is provided on a 24 hour basis, including weekends and holidays, by staff comprising veterinarians and animal health technicians. In the case of *M*. *tuberculosis* H37Ra administration, the mice were anesthetized with Nembutol anesthetic (pentobarbital sodium, 60 mg/Kg) intraperitoneally to reduce the discomfort caused by mouse intratracheal injection. Excessive CO_2_ gas were used for euthanasia. In brief, C57BL/6 mice were anesthetized, and administered intratracheally with 12 mg/Kg (about 3×10^6^ CFU for a 25 g mouse) H37Ra or 10 mg/Kg LPS in 50 μl of PBS. Twenty-four hours after intratracheal injection, the mice were sacrificed and their lung tissue was collected, then fixed in 4% (w/v) paraformaldehyde overnight at 4°C. This was followed by embedding in paraffin wax and processing to generate 5 μm sections for histological analysis. To collect the BAL fluid, the lungs of the mice were flushed twice with 800 μl PBS.

### H&E staining

Paraffin-embedded lung sections were deparaffinized and hydrated in distilled water. The lung sections were then stained with hematoxylin and eosin for 10 min and 30 sec, respectively. The quantification of histology was performed manually in a blinded fashion using Image-Pro Plus 5.0. The purple-based H&E staining for inflammatory nuclei staining was compared with the basal level present in a mock group, and a ratio percentage of positively stained area was calculated by the program.

### Ziehl-Neelsen staining

Paraffin-embedded lung sections were deparaffinized and hydrated in distilled water. The lung sections were then stained with Carbol fuchsin/Methylene blue (Sigma, 21820-1L and 03978-250ML) to detect the presence of mycobacteria according to the WHO standard protocols. In brief, the lung sections were stained with Carbol fuchsin for 5 min under a heater and then destained with 3% acid-alcohol for 2 minutes. Finally, the sections were stained with Methylene blue for 1 min and examined by microscopy using the 100x oil immersion objective.

### TUNEL assay

Paraffin-embedded lung sections were deparaffinized and hydrated in distilled water. After deparaffinization, the tissue sections were incubated with Protease K working solution (20 μg/ml Protease K in10 mM Tris-HCl, pH7.4) for 1 hour at room temperature. To examine the apoptotic tissue areas, an *In situ* Cell Death Detection Kit, POD (Roche), was used according to the manufacturer’s instructions. The quantification in tissue sections was carried out manually in a blinded fashion using Image-Pro Plus 5.0. The TUNEL-positive area was compared with the basal level present in a mock group using color threshold, and a ratio percentage of positively stained area was calculated by the program.

### Statistical analysis

Data are expressed as the mean ± SD. All statistical analyses were conducted using Prism (version 6.01). The significance differences between groups were determined by multiple t-tests (*p < 0.05; **p < 0.01).

## Results and discussion

### CLEC9A interacts with *Mycobacterium spp*.

**Fig 1:**
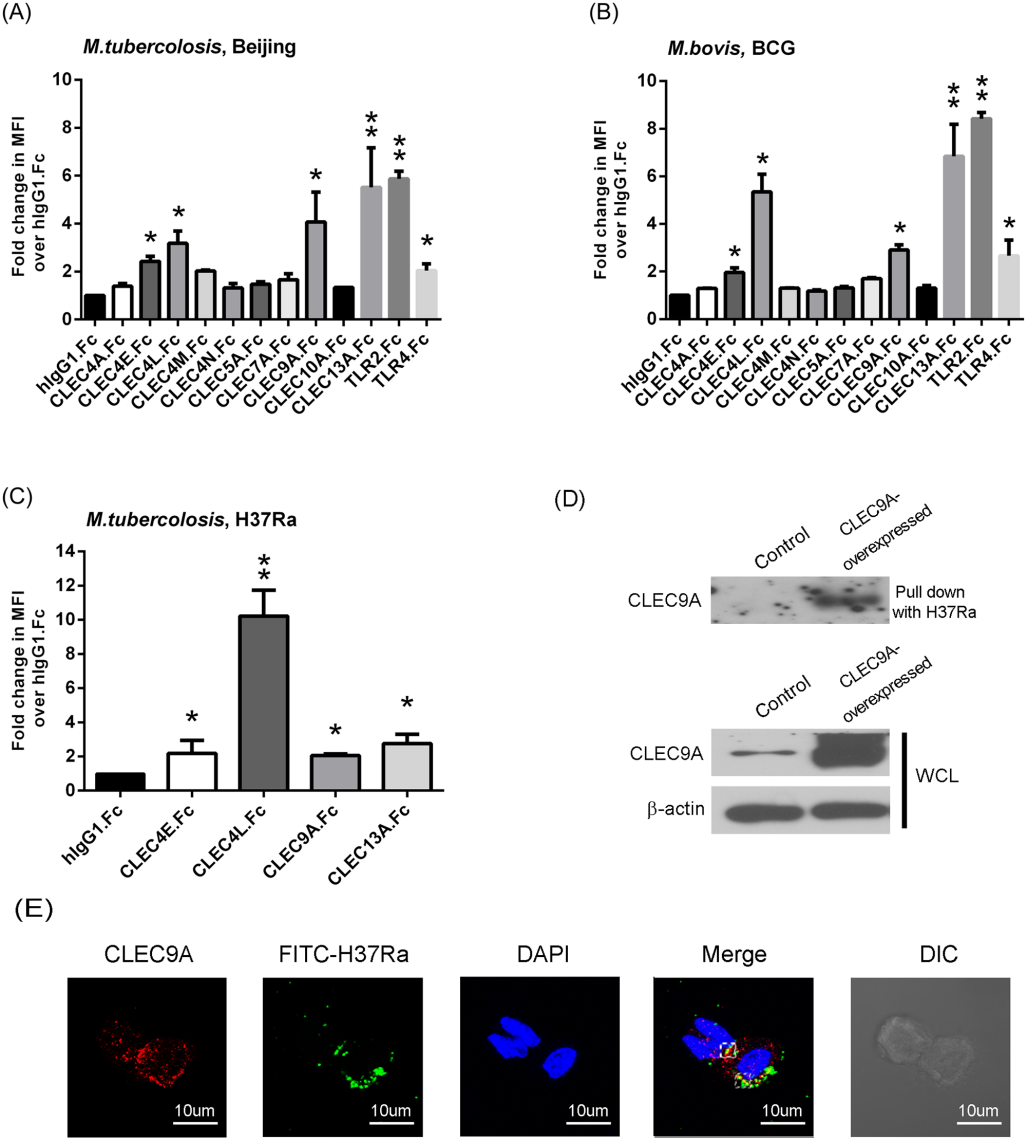
CLEC9A is identified as a novel candidate that is able to bind with mycobacteria. Interaction of heat-killed (A) *M*. *tuberculosis* Beijing, (B) *M*. *bovis* BCG and (C) *M*. *tuberculosis* H37Ra with human receptor-Fc fusion protein was determined by flow cytometry. Human IgG1 was used as a negative control. MFI, mean fluorescence intensity. Data were expressed as mean ± SD of three independent experiments. Two-tailed multiple t-tests were performed (*, *p* < 0.05; **, *p* < 0.01). (D) The pull-down assay for CLEC9A using H37Ra. H37Ra was incubated with cell lysate from control or CLEC9A-overexpressing HEK293T cells. After washing and gel-separation, the interaction of H37Ra with CLEC9A was detected by immunoblotting. (E) The membrane localization of H37Ra and CLEC9A was analyzed by confocal microscopy. The THP-1 cells were incubated with FITC-labeled H37Ra for 1 hour, fixed, and stained with antibody against CLEC9A and DAPI. Representative confocal images of three independent experiments is shown.

CLRs that are mainly expressed on myeloid cells were screened for their ability to bind to heat-killed *Mycobacterium* spp., namely *M*. *tuberculosis* Beijing and *M*. *bovis* BCG, using an *in vitro* lectin binding assay (Fig 1A and 1B, S1 Fig) [24]. Using human IgG1 as a control, and comparing these results with the increase in mean fluorescence intensity (MFI) by flow cytometry analysis, CLEC4L (DC-SIGN), CLEC4M (L-SIGN), CLEC4E (Mincle), TLR2 and TLR4 were found to have positive binding; all of these proteins have been reported to interact with mycobacteria in previous studies [36, 37]. Interestingly, CLEC4M (L-SIGN) was shown to bind with *M*. *tuberculosis* Beijing, but not *M*. *bovis* BCG. Furthermore, in addition to the above proteins, two novel CLRs, CLEC9A (DNGR-1), and CLEC13A (BIMLEC), were identified as able to bind with mycobacteria (Fig 1A and 1B, S1 Fig). The CLRs that showed positive binding with *M*. *tuberculosis* Beijing, and *M*. *bovis* BCG, namely CLEC4L, CLEC4E, CLEC9A, and CLEC13A, were then confirmed to also interact with *M*. *tuberculosis* H37Ra (Fig 1C). In a preliminary study, CLEC13A has no effect on the mycobacteria-mediated production of cytokines and chemokines (S2 Fig). Therefore, the study was focused on the role of CLEC9A.

Furthermore, it must be noted that the heat-killed mycobacteria might change the carbohydrates and lipid composition in the, leading to the loss of surface carbohydrates or the exposure of inner cell walls. In the *in vitro* lectin binding assay (Fig 1), CLEC7A (Dectin-1), unlike a report by Yadav and Schorey using viable mycobacteria [20], did not show any significant interaction with mycobacteria, which might due to the alteration of mycobacterial cell surface by heat-inactivation. To address this concern, heat-killed and viable *Mycobacteria kansasii* were used for CLEC9A binding assay. The data showed that CLEC9A is able to bind with heat-killed or viable *M*. *kansasii* with in vitro lectin binding assay (S3 Fig), indicating that the interaction between CLEC9A and mycobacteria was not abolished by the heat-killing procedure. Moreover, the binding of CLEC9A with mycobacteria is not resulted from the molecules exposed from inner cell walls.

CLEC9A is mainly expressed on myeloid lineage cells, in particular BDCA3+ dendritic cells [28]. The mRNA of CLEC9A in human monocytic THP-1 cells and human peripheral blood mononuclear cells (PBMC) from healthy donors was examined (S4A Fig). The results show that CLEC9A can be detected in THP-1 cells and human PBMCs. However, the CLEC9A mRNA level in THP-1 cells did not change in response to H37Ra (S4B Fig). The binding of mycobacteria H37Ra with CLEC9A was further examined by pull-down assay and confocal microscopy analysis. H37Ra was incubated with cell lysate from control or CLEC9A-overexpressed HEK293T cells. The binding of H37Ra and CLEC9A was analyzed by immunoblotting. The results suggest that CLEC9A is able to be pull-down by H37Ra (Fig 1D). Moreover, based on confocal microscopy analysis, FITC-labeled H37Ra can be seen to be co-localize with CLEC9A on the membrane (Fig 1E).

Since CLEC9A has been reported as an endocytic receptor, the impact of CLEC9A on the binding of *M*. *tuberculosis* with macrophages was examined. CLEC9A was knocked-down using a lentiviral-based shRNA system, and the knock down efficiency of CLEC9A in THP-1 cells was confirmed by examining the mRNA and protein levels in the knocked-down cells. shRNA#1 and shRNA#3 were chosen for the following experiments. By incubating THP-1 cells with FITC-modified H37Ra for 2 hours at 4°C, the FITC-positive cells, which indicate the binding of mycobacteria, could be detected by flow cytometry. As shown in S5 Fig, there was no significant difference between control and the CLEC9A-silenced cells, indicating that the interaction between *M*. *tuberculosis* and CLEC9A is not required for host-pathogen binding.

### Mycobacteria-induced SYK and MAPK activation was decreased in CLEC9A-knocked down macrophages

**Fig 2:**
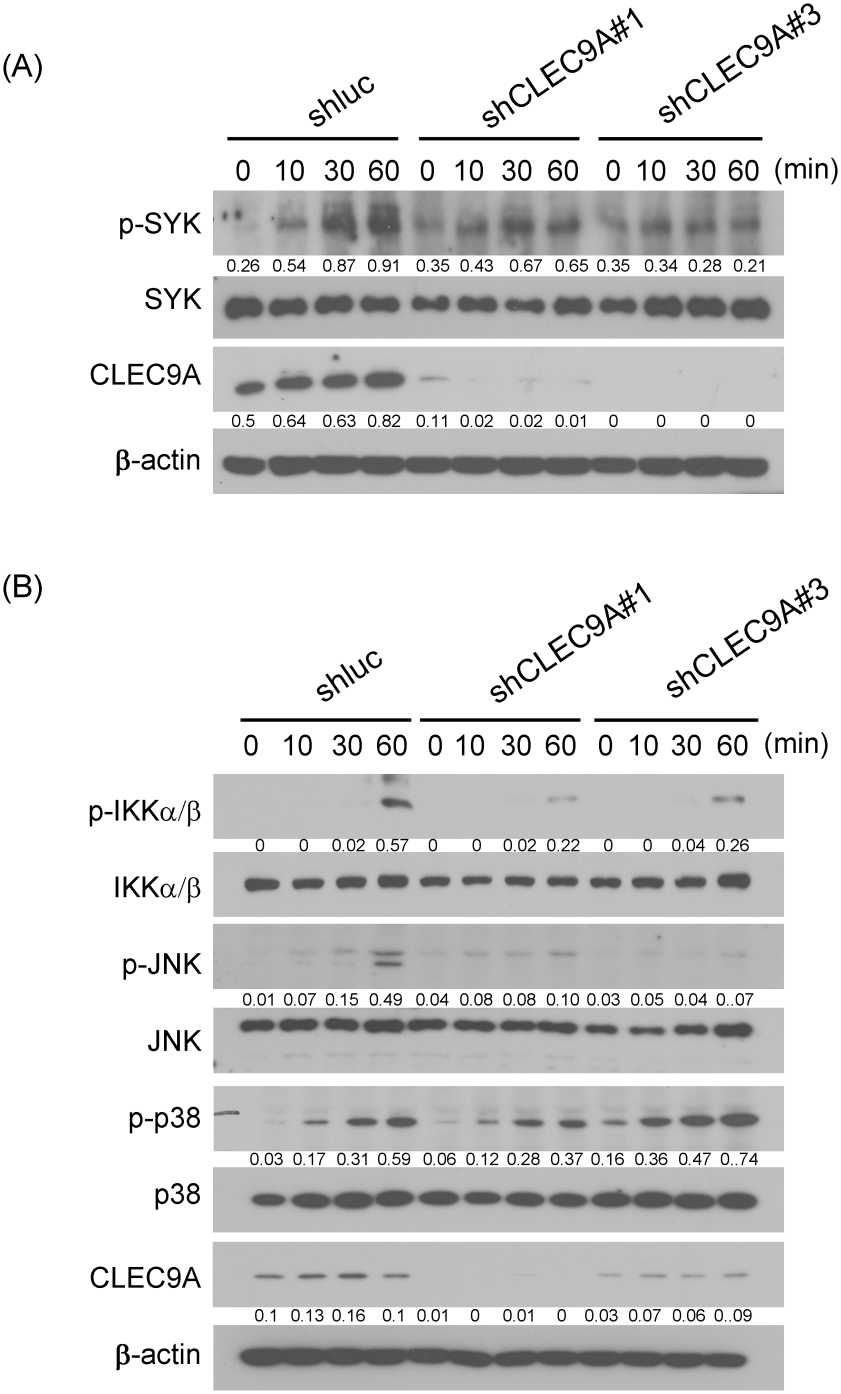
Activation of SYK and MAPK is decreased in CLEC9A-knocked down cells that had been H37Ra stimulated. CLEC9A in THP-1 cells was knocked down using lentiviral-based shRNA. After treating with heat-killed H37Ra for the indicated times, the cells were lysed and subjected to immunoblot analysis. (A) Syk activation was analyzed by measuring protein phosphorylation using the indicated specific antibodies. (B) MAPK signal activation was analyzed by measuring protein phosphorylation using the indicated specific antibodies. Representative data from three independent experiments are showed. The western blots were quantified with densitometry, the relative amount normalized to β-actin was calculated. The densitometry data was presented as mean values from three experiments.

CLR signaling has been indicated to activate SYK and various downstream effectors, including MAPKs and NF-κB [38]. Although CLEC9A did not affect the binding ability of *M*. *tuberculosis* with macrophages, the role of CLEC9A in signal activation in response to H37Ra was investigated (Fig 2). The phosphorylation of SYK was reduced in CLEC9A-knocked down THP-1 cells in response to H37Ra treatment. Moreover, the decrease in SYK activation was further investigated by examining the downstream signaling. As shown in Fig 2B, the IKK and JNK pathways, which responded to H37Ra treatment, were also inhibited in the CLEC9A-knocked down THP-1 cells.

#### CLEC9A is critical to the induction of CXCL8 and IL-1β in macrophages that have been treated with mycobacteria

**Fig 3:**
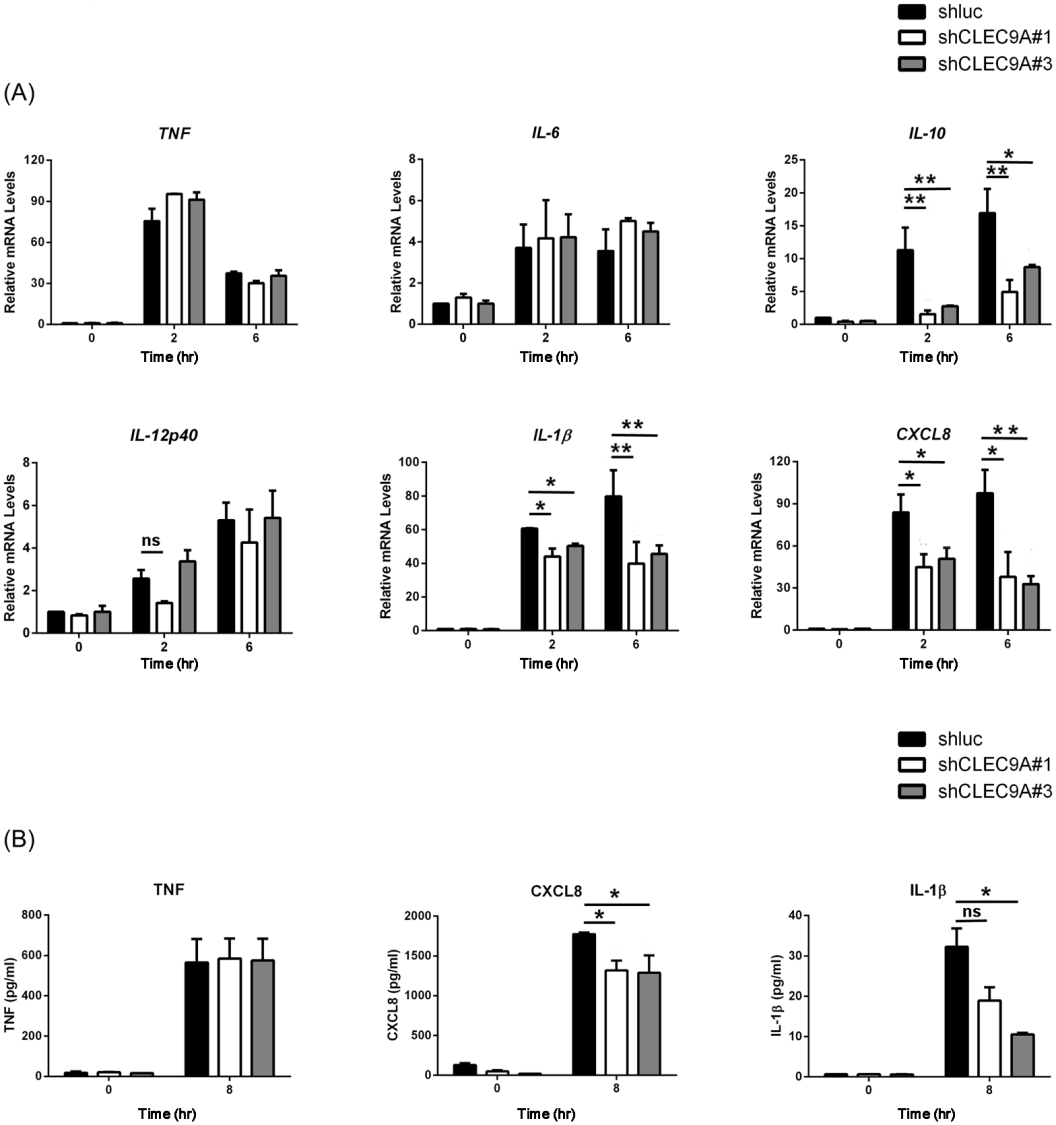
Induction of CXCL8 and IL-1β is decreased in CLEC9A-silenced THP-1 cells treated with H37Ra. Control and CLEC9A-silenced THP-1 cells were stimulated with heat-killed H37Ra for the indicated times. (A) Total RNA was extracted using TRIzol. After reverse transcription, the expression levels of the indicated mRNAs were measured by Q-PCR and normalized against GAPDH mRNA. The relative mRNA level in knock-down control THP-1 cells (shluc) at 0 hour was set as 1.0. (B) The medium was collected and analyzed for TNF, CXCL8 and IL-1β level by ELISA. The results are mean ± SD of three separate experiments. Two-tailed multiple t-tests were performed (*, *p* < 0.05; **, *p* < 0.01).

Since CLEC9A is involved in the mycobacteria-mediated activation of SYK, JNK and p38, the production of cytokines and chemokines regulated by CLEC9A was examined. In CLEC9A-silenced THP-1 cells treated with H37Ra, induction of IL-10, IL-1β and CXCL8 (also known as IL-8) mRNA was decreased (Fig 3A). However, the mRNA levels of various inflammatory cytokines, including TNF, IL-6 and IL-12, were not affected by CLEC9A silencing. In a manner that correlated with the mRNA expression level results, the protein levels of CXCL8 and IL-1β, but not of TNF, were decreased in the H37Ra treated CLEC9A-knocked down THP-1 cells (Fig 3B).

**Fig 4:**
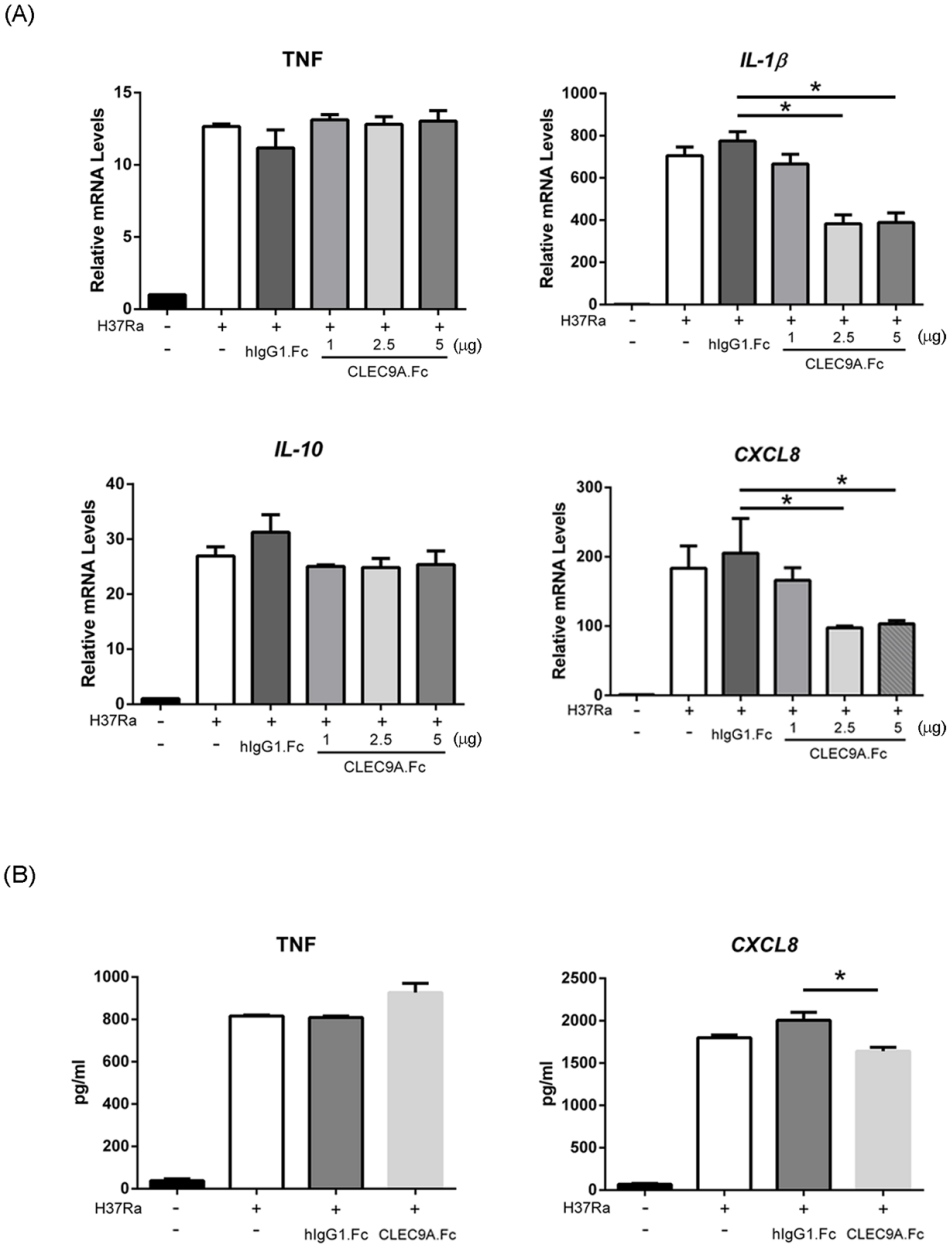
Induction of CXCL8 in THP-1 cells was decreased in response to H37Ra in the presence of CLEC9A blocked. (A) Heat-killed H37Ra was pre-incubated with human IgG1 control or CLEC9A-Fc fusion protein (0, 1 or 5 μg) for 30 min. After washing, THP-1 cells were treated with H37Ra for 6 hours. Total RNA was extracted using TRIzol. After reverse transcription, expression of the indicated mRNAs was measured by Q-PCR and normalized against GAPDH mRNA. The relative mRNA level in the medium control (M) was set as 1.0. (B) Heat-killed H37Ra was pre-incubated with human IgG1 control or CLEC9A-Fc fusion protein (5 μg) for 30 min. The medium from control or THP-1 cells that had been treated with H37Ra for 8 hours were collected and analyzed to measure their TNF and CXCL8 levels by ELISA. The results are mean ± SD of three separate experiments. Two-tailed multiple t-tests were performed (*, *p* < 0.05).

Next, human CLEC9A-Fc fusion protein was used to block the specific surface molecule in mycobacteria that can be recognized and bound by macrophage CLEC9A. After pre-incubation of H37Ra with human CLEC9A-Fc, we found that IL-1β and CXCL8 mRNA induction was decreased. However, the mRNA levels of TNF, IL-6 and IL-12 did not change when H37Ra had bound (Fig 4A). The protein level of CXCL8, but not TNF, was also decreased (Fig 4B). Surprisingly, unlike the results with the CLEC9A silencing, there was no difference in the induction of IL-10 mRNA with or without CLEC9A-Fc blocking (Fig 4A).

Interestingly, by pre-treating THP-1 cells with the SYK inhibitor, BAY 61–3606, the production of various cytokines or chemokines, including TNF, IL-6, IL-10, CXCL8, and IL-1β, was inhibited in response to H37Ra (S6 Fig). These results suggest that the SYK pathway is critical to the mycobacteria-mediated production of cytokines/chemokines. However, it has been reported that mycobacteria interact with multiple receptors that are able to activate SYK signaling, such as DC-SIGN and Mincle; thus, it is possible that CLEC9A contributes only to part of the SYK activation. Taken as a whole, CLEC9A may act as a modulator that controls the induction of specific cytokines, such as CXCL8 and IL-1β.

### The role of mycobacteria and CLEC9A in macrophage-modulated neutrophil migration and activation

**Fig 5:**
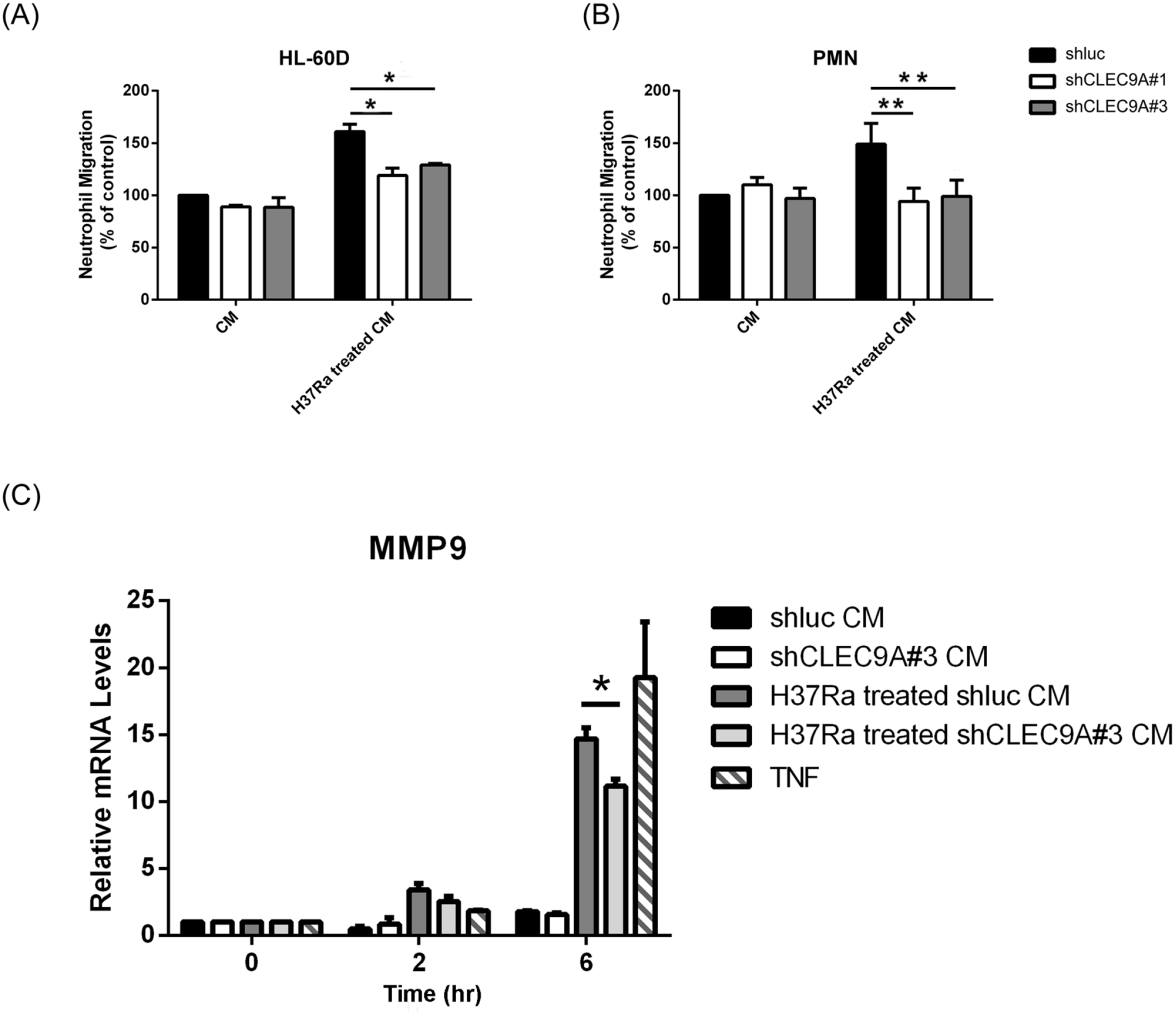
Neutrophil migration and activation declined in response to CLEC9A-knocked down macrophages that had undergone H37Ra stimulation. The neutrophil migration levels of (A) HL-60D cells and (B) human primary PMNs were assayed using a multiwell chamber chemotaxis assay. Neutrophils were resuspended in the upper chamber of a Transwell plate and conditioned medium was added to the lower chamber. Conditioned medium was obtained from control or CLEC9A-knocked down THP-1 cells that had been treated with heat-killed H37Ra for the indicated times. Only after removal of H37Ra by centrifugation was the conditioned medium used for the migration assays. Following incubation for 90 min, the neutrophils that had migrated through Transwell membrane were counted. (C) HL-60D cells were incubated with conditioned medium from knock-down control or CLEC9A-silencing THP-1 cells with or without H37Ra treatment for the indicated times. Total RNA was extracted, reverse transcribed, and the expression of the MMP9 mRNA was measured by Q-PCR and normalized against GAPDH mRNA. The relative mRNA level in the cells incubated with conditioned medium from knock-down control THP-1 cells without H37Ra treatment (shluc CM) at 0 hour was set as 1.0. Data are mean ± SD of three separate experiments. Two-tailed multiple t-tests were performed (*, *p* < 0.05; **, *p* < 0.01).

Both IL-1β and CXCL8 have been shown to be chemokines that are crucial to neutrophil recruitment [39]. Therefore, the impact of CLEC9A-regulated CXCL8 production in neutrophil migration and activation was investigated. Neutrophil-like differentiated HL-60 (HL-60D) cells and primary human neutrophils were used. Migration assays were conducted using a 24-well Transwell system with HL-60D or human neutrophils in the upper well, and conditioned medium in the lower well. As shown in Fig 5A and 5B, the migration of HL-60D cells and primary human neutrophils were decreased in response to the presence of conditioned medium from CLEC9A-knocked down THP-1 cells treated with H37Ra. Moreover, the activation of neutrophils induced by conditioned medium was detected via MMP9 expression. The results showed that the induction of MMP9 mRNA in neutrophils was decreased in the presence of medium from CLEC9A-silenced THP-1 cells (Fig 5C).

### CLEC9A signaling is required for cell infiltration *in vivo*

**Fig 6:**
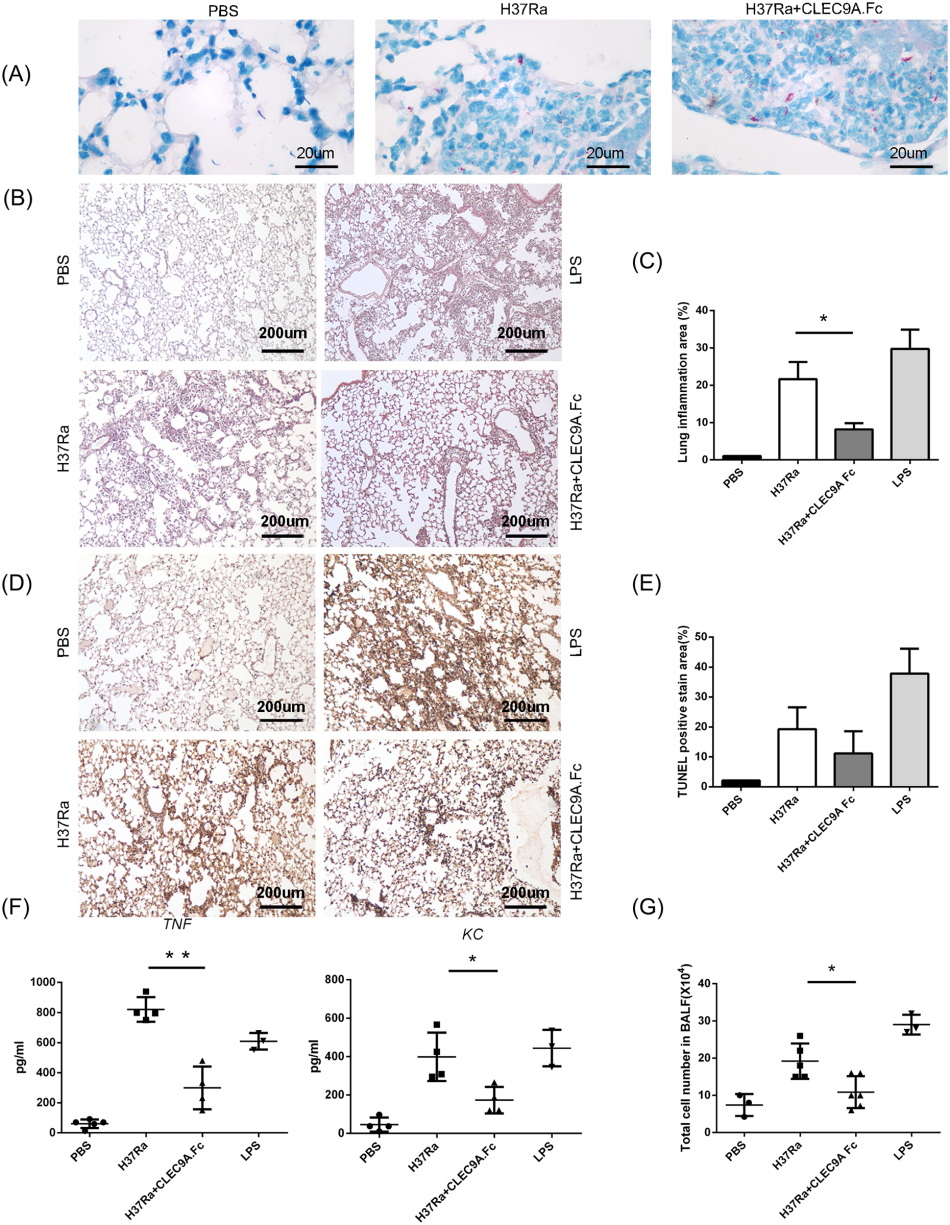
Leukocyte infiltration *in vivo* is decreased by CLEC9A inhibition with H37Ra. C57BL/6 mice were infected intratracheally with heat-killed H37Ra with or without pre-incubation with human CLEC9A-Fc fusion protein. The mice were sacrificed 24 hours post-H37Ra challenge. **(A)** Formalin-fixed, paraffin embedded lung sections were stained for acid-fast bacteria by the Ziehl-Neelsen method. 1000× magnification, and **(B)** hematoxylin and eosin (H&E)-stained lung histology was examined. 100× magnification. Representative data of three independent experiments are shown. **(C)** The quantification of lung infiltration using H&E staining. **(D)** The amount of apoptosis present was determined by TUNEL stain using sections of lung. 100× magnification. Representative data from three independent experiments are shown. **(E)** The quantification of cell death by TUNEL staining. **(F)** The amounts of TNF and CXCL8 in bronchoalveolar lavage (BAL) fluid were analyzed by ELISA. **(G)** The total cell number was counted in the cytocentrifuged BAL fluid samples. The data points represent values from n  =  3–6 mice per group. Two-tailed multiple t-tests were performed (*, *p* < 0.05; **, *p* < 0.01).

Next, to validate the significance of the results from the *in vitro* experiments, the role of CLEC9A in macrophages *in vivo* was investigated. C57BL/6 mice were exposed intratracheally to H37Ra with or without the presence of human CLEC9A-Fc fusion protein and then sacrificed 24 hours post-infection. Lung tissue was subjected to histological analysis and bronchoalveolar lavage (BAL) fluid was processed to detect the amount of cytokines and chemokines present. Furthermore, the total number of cells present in BAL fluid was counted. Using the Ziehl-Neelsen method to stain mycobacteria, the result showed that CLEC9A-Fc fusion protein did not interrupt the entry of H37Ra into the lung (Fig 6A). Using H&E staining, it was found that lung inflammation and cell infiltration was decreased when H37Ra with CLEC9A-Fc fusion protein pre-incubation was used compared to the control without CLEC9A-Fc fusion protein (Fig 6B and 6C). TUNEL assays measuring cell death showed a marginal, but not statistical significant, decrease in lung damage for the CLEC9A-Fc pre-incubated H37Ra samples (Fig 6D and 6E). Cytokine production and cell infiltration were also investigated. In BAL fluid, the level of CXCL1 (also known as KC), the murine homolog of CXCL8, and TNF were decreased in the CLEC9A-Fc pre-incubated H37Ra-treated samples (Fig 6F). Moreover, the total cell numbers in the BAL fluid were decreased about 50%, which suggests less cell infiltration (Fig 6G). The decrease in TNF, which was different from the results obtained using THP-1 cells in vitro, might be due to less infiltration of immune cells into the BAL fluid.

## Conclusion

We identified CLEC9A as a novel CLR that interacts with mycobacteria, and demonstrated that CLEC9A is involved in SYK and MAPK activation in response to H37Ra stimulation. By using heat-killed and viable *Mycobacteria kansasii* for CLEC9A binding assay, we showed that the interaction between CLEC9A and mycobacteria was not altered by the process of heat-killing. Using CLEC9A-knocked down THP-1 cells, the CXCL8 and IL-1β production induced by heat-inactivated H37Ra was found to be selectively decreased. Using CLEC9A-Fc fusion protein to block the interaction between mycobacteria and CLEC9A on macrophages, similar results to those obtained using CLEC9A-silenced cells were observed. Furthermore, CLEC9A-regulated CXCL8 and IL-1β production by macrophages leads to a modulation of neutrophil migration and activation. In a murine model, CLEC9A-Fc treatment is able to decrease H37Ra-mediated leukocyte infiltration and lung inflammation at an early stage. These findings with respect to CLEC9A during mycobacteria infection might have potential therapeutic applications in terms of the regulation of the specific cytokines and chemokines that modulate macrophage-mediated neutrophil recruitment.

CLEC9A has been reported to recognize the actin filaments of damaged cells, a damage-associated molecular pattern (DAMP), and thus is as a receptor for necrotic cells [25, 40]. However, ligands as pathogen-associated molecular patterns (PAMP) that can be recognized by CLEC9A have not yet been identified. Based on our study, which shows the interaction between CLEC9A and mycobacteria, the cell wall components of mycobacteria might serve as PAMPs for CLEC9A recognition. Although we have demonstrated that the engagement of mycobacteria with CLEC9A regulates activation of the SYK signal and induces various effector genes, CLEC9A signaling is also activated by a number of endogenous ligands such as actin filaments from dead cells [25, 40]. It is known that mycobacteria infection does induce the necrosis of host cells. Therefore, it is possible that both mycobacterial PAMPs and DAMPs from dead cells are both able to contribute to the CLEC9A-modulated immune responses after mycobacteria administration *in vitro* and *in vivo*.

So far, TLRs, including TLR2, and TLR4, have been shown to predominantly regulate anti-mycobacterial immunity [41]. On the other hand, CLRs, such as DC-SIGN and Mincle, function as the modulators during mycobacteria infection. DC-SIGN modulates mycobacteria-induced TLR signaling in human DCs by up-regulating the production of the immunosuppressive cytokine IL-10 [42]. Mincle promotes mycobacteria-induced TLR2 signaling pathways synergistically in neutrophils [43]. Based on our study, the interaction between CLEC9A and mycobacteria is not required for the binding of macrophages with mycobacteria, which suggests that multiple PRRs interact with mycobacteria [9] and only some of these PRRs play critical roles in binding during the recognition stage. Moreover, the selective up-regulation of IL-1β and CXCL8 through the SYK and MAPK pathways, indicates that CLEC9A is a specialized receptor linked to the regulation the mycobacteria-mediated immune response.

Although the role of neutrophils in *M*. *tuberculosis* infection remains controversial, the recruitment of neutrophils following infection is an important physiological response. Most studies suggest that mycobacterial-infected macrophages secrete cytokines and chemokines that allow neutrophil recruitment and activation [44]. Yang et al. demonstrated that neutrophil recruitment into granulomas is mediated by signals from dying infected macrophages using a zebrafish model [45]. The identification of CLEC9A might account for both types of mechanism during neutrophil accumulation after mycobacteria infection. Thus, it might act as a receptor that interact directly with mycobacteria or, alternatively, as a receptor that senses cell death. In macrophages, CLEC9A selectively regulates IL-1β and CXCL8, both of which are critical to neutrophil recruitment.

CLEC9A in DCs is involved in cross-priming cytotoxic T lymphocytes (CTLs) against the antigens of dead cells by transporting necrotic cell cargo into an endosomal recycling compartment [21]. As we have found that CLEC9A interacts with mycobacteria, it is possible that CLEC9A in DCs or macrophages might also be able to induce CTLs against the antigens of mycobacteria. Whether or not CLEC9A mediates phagocytosis and endosomal translocation of mycobacteria needs to be further investigated.

In summary, our study provides insights into CLEC9A, a specialized receptor that interacts with *M*. *tuberculosis* and is involved in SYK activation. CLEC9A-mediated SYK activation specifically promotes the production of CXCL8 and IL-1β, which are central to neutrophil recruitment and activation during the early stage of infection. The biological findings explored in the study increase our understanding of *M*. *tuberculosis* recognition and the receptor-pathogen mediated immune response, and it is hoped they will help with future therapy and vaccination design.

## Supporting information

S1 Fig *In vitro* lectin-binding assay using flow cytometry. Interaction of *M*. *tuberculosis* Beijing with human receptor-Fc fusion proteins was determined by flow cytometry. Human IgG1 was used as the negative control. MFI, mean fluorescence intensity. Representative data from three independent experiments are shown.
(TIF)

S2 Fig CLEC13A-regulated cytokine production in human THP-1 cells with H37Ra treatment. Control and CLEC13A-silenced THP-1 cells were stimulated with heat-killed H37Ra for the indicated times. Total RNA was extracted using TRIzol. After reverse transcription, the expression levels of mRNAs were measured by Q-PCR and normalized against GAPDH mRNA. (A) The expression of CLEC13A (BIMLEC) mRNA level. (B) The mRNA expression levels of TNF, IL-10, IL-1β and CXCL8. The relative mRNA level in knock-down control THP-1 cells (shluc) at 0 hour was set as 1.0. The results are mean ± SD of three separate experiments. Two-tailed multiple t-tests were performed (*, *p* < 0.05; **, *p* < 0.01).
(TIF)

S3 Fig CLEC9A is able to bind with heat-killed and viable *Mycobacterium kansasii*. Interaction of heat-killed and viable *Mycobacterium kansasii* with human receptor-Fc fusion protein was determined by flow cytometry. Human IgG1 was used as a negative control, and heat-killed H37Ra was used as a positive control. MFI, mean fluorescence intensity. Data were expressed as mean ± SD of three independent experiments. Two-tailed multiple t-tests were performed (*, *p* < 0.05).
(TIF)

S4 Fig Expression patterns of CLEC9A in human THP-1 cells. The expression of CLEC9A was analyzed by Q-PCR to measure mRNA levels. (A) Human THP-1 cells were subjected to macrophage-like differentiation by PMA treatment for 2 days. PBMCs were isolated from the whole blood of healthy human donors. The cells were collected and mRNA was extracted. (B) CLEC9A mRNA expression level in THP-1 cells in response to H37Ra. The cells were treated with H37Ra for the indicated times. mRNA was extracted and subjected to Q-PCR analysis. Two-tailed multiple t-tests were performed (*, *p* < 0.05; **, *p* < 0.01).
(TIF)

S5 Fig CLEC9A silencing does not interfere with the binding of *M*. *tuberculosis* to macrophages. Human THP-1 cells with or without CLEC9A silencing were treated with FITC-labeled mycobacteria for two hours at 4°C. After washing, at least 100 cells per slide were counted by fluorescence microscopy to obtain the percentage of FITC-positive macrophages. Results are mean ± SD of three separate experiments. Two-tailed multiple t-tests were performed (*, *p* < 0.05; **, *p* < 0.01).
(TIF)

S6 Fig SYK inhibitor decreases the induction of cytokines and chemokines in THP-1 cells that have undergone H37Ra engagement. THP-1 cells were pretreated with the SYK inhibitor, BAY 61–3606, for 30 min and then stimulated with H37Ra for the indicated times. Total RNA was extracted using TRIzol. After reverse transcription, the expression of the indicated mRNAs was measured by Q-PCR. Results are mean ± SD of three separate experiments. Two-tailed multiple t-tests were performed (*, *p* < 0.05; **, *p* < 0.01).
(TIF)
