# Supplementary figures and images for: CLEC9A modulates macrophage-mediated neutrophil recruitment in response to heat-killed Mycobacterium tuberculosis H37Ra
Source: PLoS One. 2017 Oct 24;12(10):e0186780. doi: 10.1371/journal.pone.0186780 (PMC5655532; doi:10.1371/journal.pone.0186780)

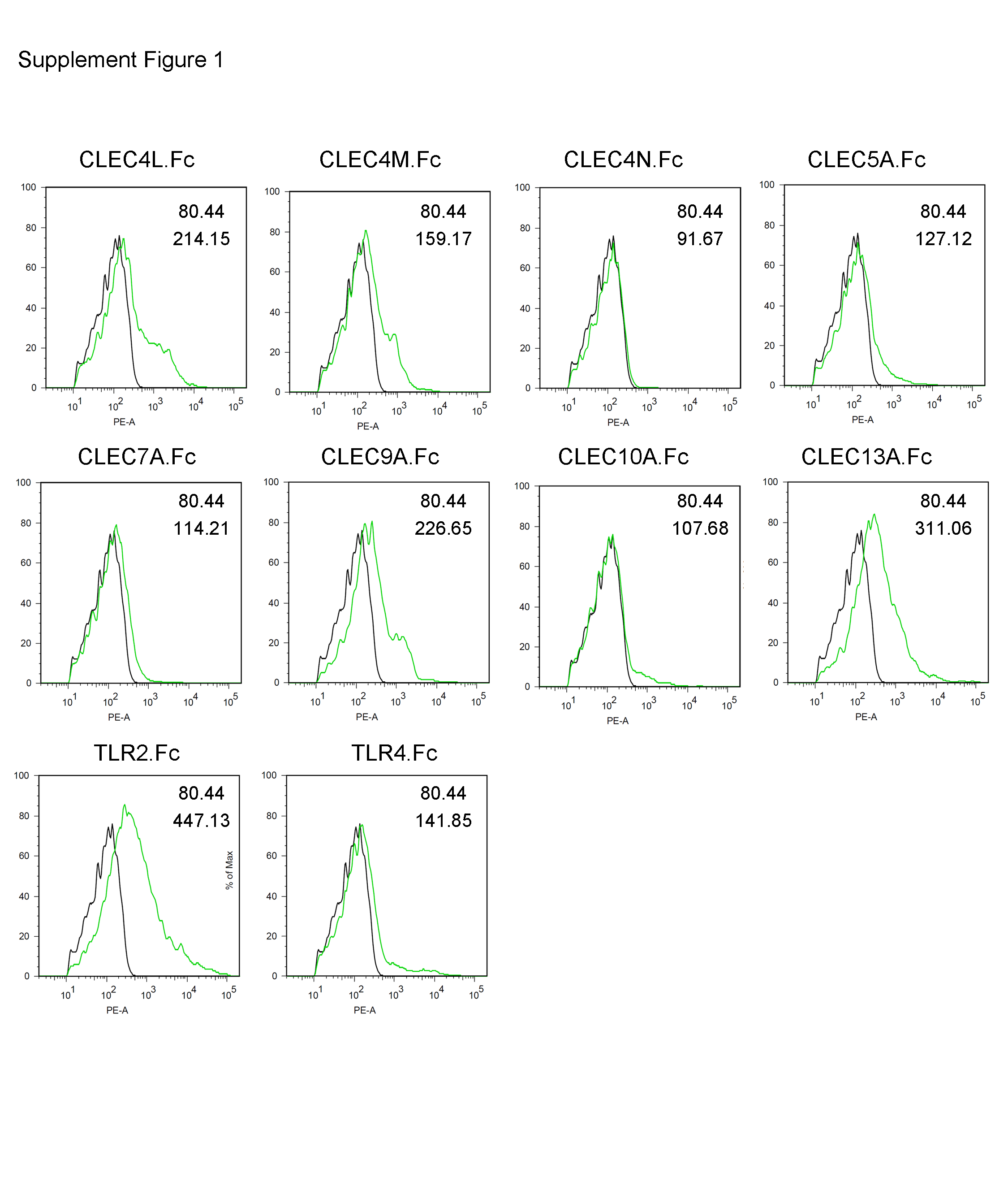

Supplement: S1 Fig — Interaction of M. tuberculosis Beijing with human receptor-Fc fusion proteins was determined by flow cytometry. Human IgG1 was used as the negative control. MFI, mean fluorescence intensity. Representative data from three independent experiments are shown. (TIF) [file pone.0186780.s001.tif]

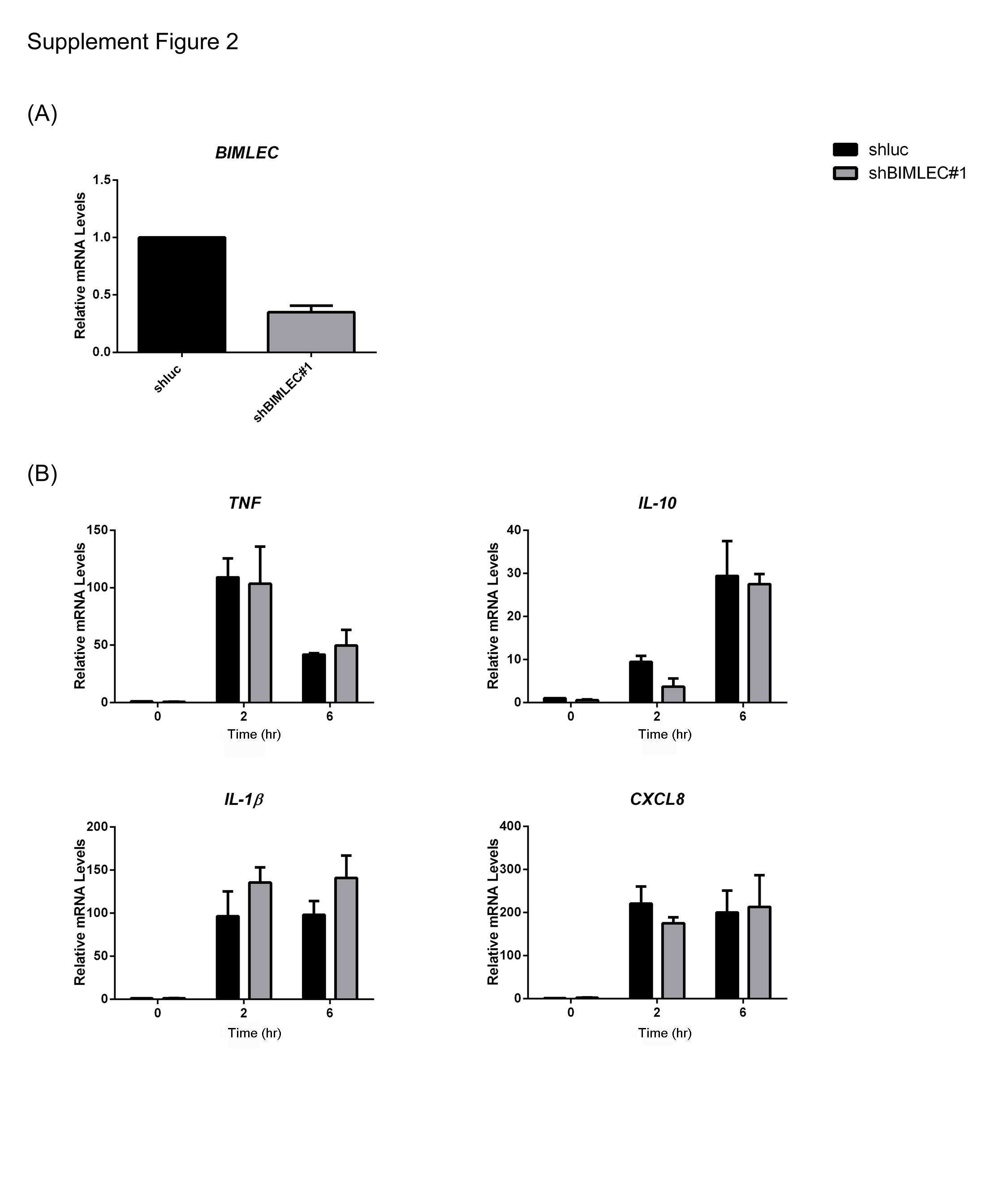

Supplement: S2 Fig — Control and CLEC13A-silenced THP-1 cells were stimulated with heat-killed H37Ra for the indicated times. Total RNA was extracted using TRIzol. After reverse transcription, the expression levels of mRNAs were measured by Q-PCR and normalized against GAPDH mRNA. (A) The expression of CLEC13A (BIMLEC) mRNA level. (B) The mRNA expression levels of TNF, IL-10, IL-1β and CXCL8. The relative mRNA level in knock-down control THP-1 cells (shluc) at 0 hour was set as 1.0. The results are mean ± SD of three separate experiments. Two-tailed multiple t-tests were performed (*, p < 0.05; **, p < 0.01). (TIF) [file pone.0186780.s002.tif]

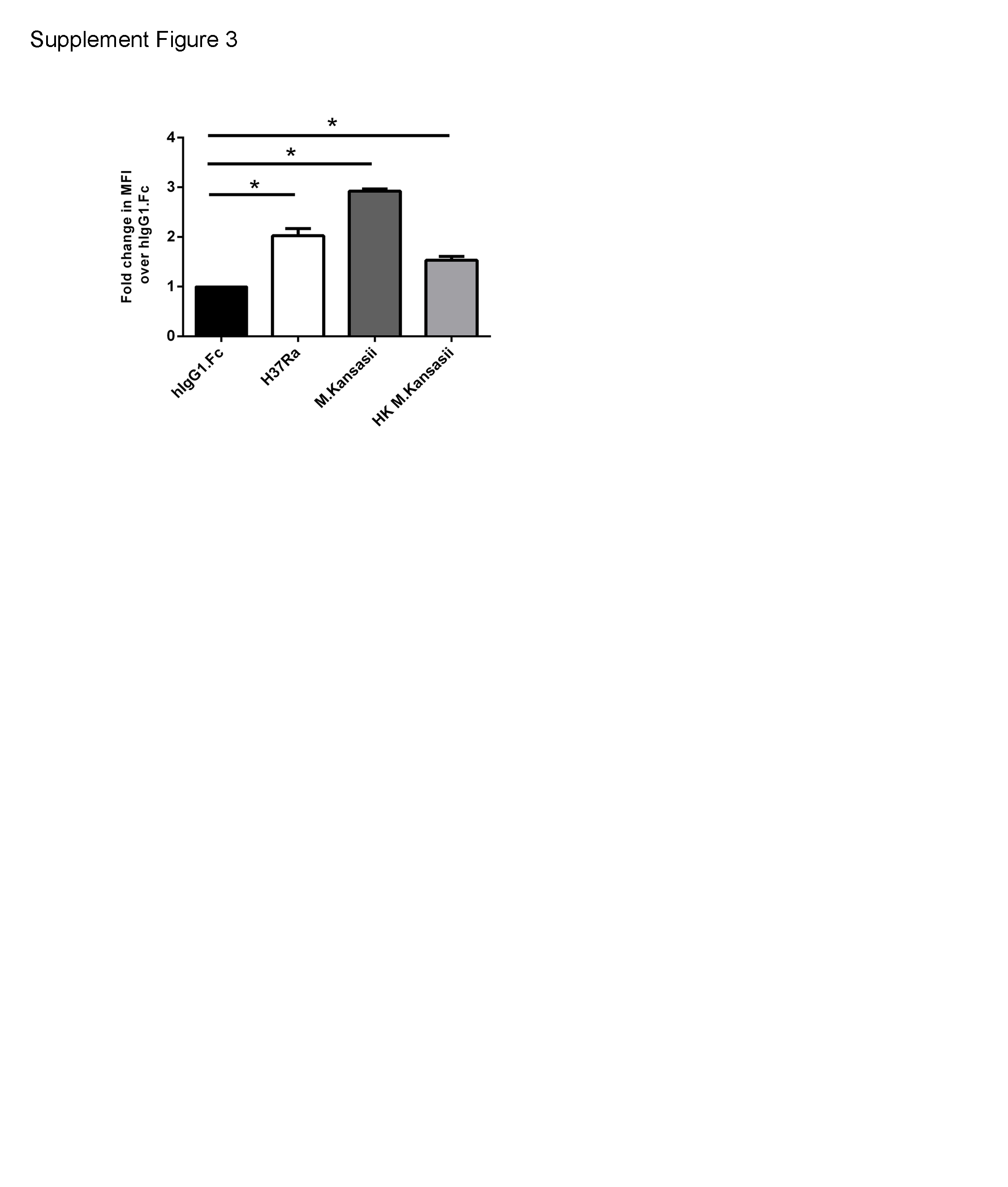

Supplement: S3 Fig — Interaction of heat-killed and viable Mycobacterium kansasii with human receptor-Fc fusion protein was determined by flow cytometry. Human IgG1 was used as a negative control, and heat-killed H37Ra was used as a positive control. MFI, mean fluorescence intensity. Data were expressed as mean ± SD of three independent experiments. Two-tailed multiple t-tests were performed (*, p < 0.05). (TIF) [file pone.0186780.s003.tif]

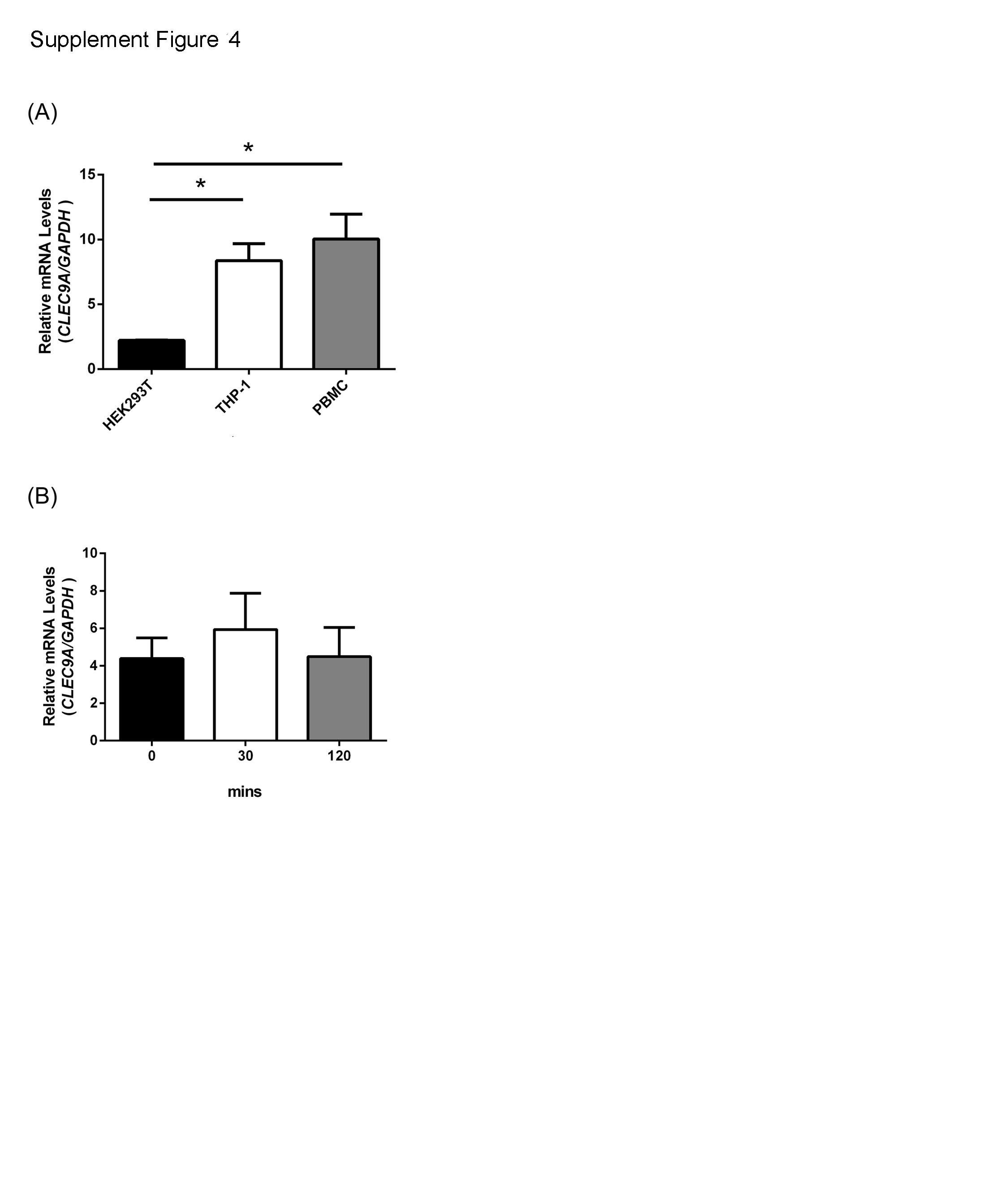

Supplement: S4 Fig — The expression of CLEC9A was analyzed by Q-PCR to measure mRNA levels. (A) Human THP-1 cells were subjected to macrophage-like differentiation by PMA treatment for 2 days. PBMCs were isolated from the whole blood of healthy human donors. The cells were collected and mRNA was extracted. (B) CLEC9A mRNA expression level in THP-1 cells in response to H37Ra. The cells were treated with H37Ra for the indicated times. mRNA was extracted and subjected to Q-PCR analysis. Two-tailed multiple t-tests were performed (*, p < 0.05; **, p < 0.01). (TIF) [file pone.0186780.s004.tif]

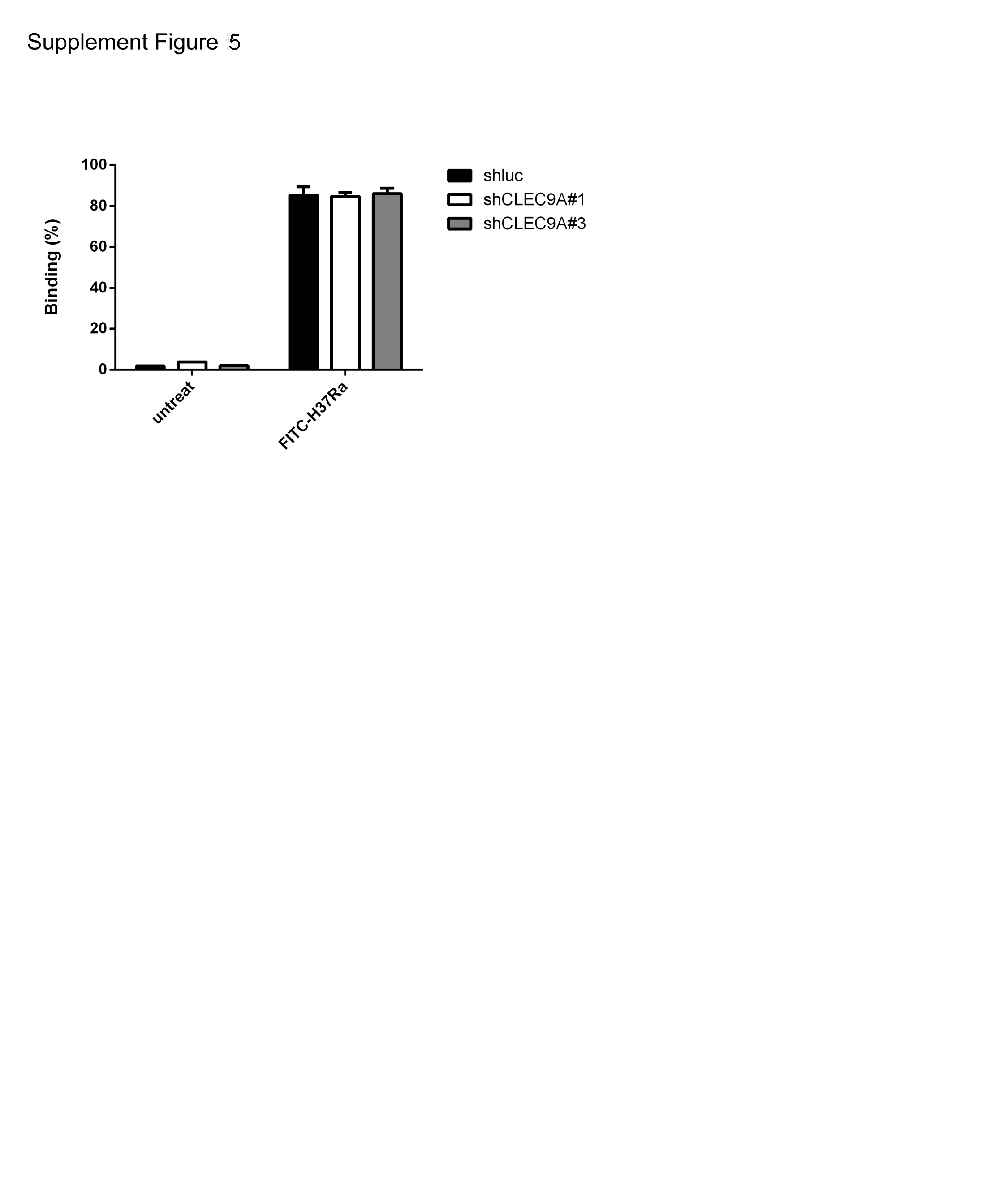

Supplement: S5 Fig — Human THP-1 cells with or without CLEC9A silencing were treated with FITC-labeled mycobacteria for two hours at 4°C. After washing, at least 100 cells per slide were counted by fluorescence microscopy to obtain the percentage of FITC-positive macrophages. Results are mean ± SD of three separate experiments. Two-tailed multiple t-tests were performed (*, p < 0.05; **, p < 0.01). (TIF) [file pone.0186780.s005.tif]

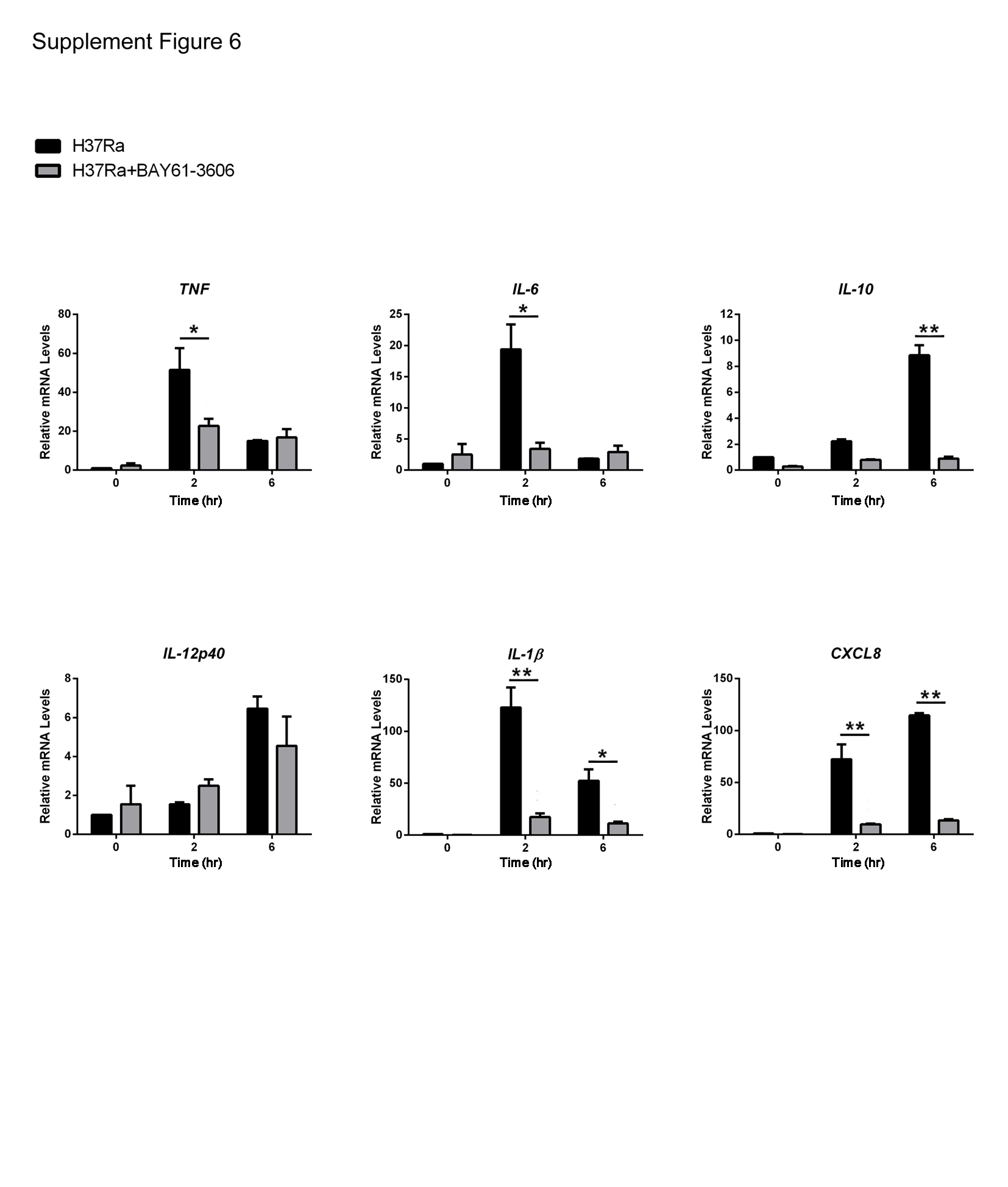

Supplement: S6 Fig — THP-1 cells were pretreated with the SYK inhibitor, BAY 61–3606, for 30 min and then stimulated with H37Ra for the indicated times. Total RNA was extracted using TRIzol. After reverse transcription, the expression of the indicated mRNAs was measured by Q-PCR. Results are mean ± SD of three separate experiments. Two-tailed multiple t-tests were performed (*, p < 0.05; **, p < 0.01). (TIF) [file pone.0186780.s006.tif]
